# Supplementary material for: Role of reactive oxygen species and isoflavonoids in soybean resistance to the attack of the southern green stink bug
Source: PeerJ. 2020 Sep 17;8:e9956. doi: 10.7717/peerj.9956 (PMC7502232; doi:10.7717/peerj.9956)
Supplement: Supplemental Information 1 — Chromatograms for external standars and examples of soybean seeds extracts chomatograms. [file peerj-08-9956-s001.docx]

**CHROMATOGRAMS FOR EXTERNAL STANDARDS**


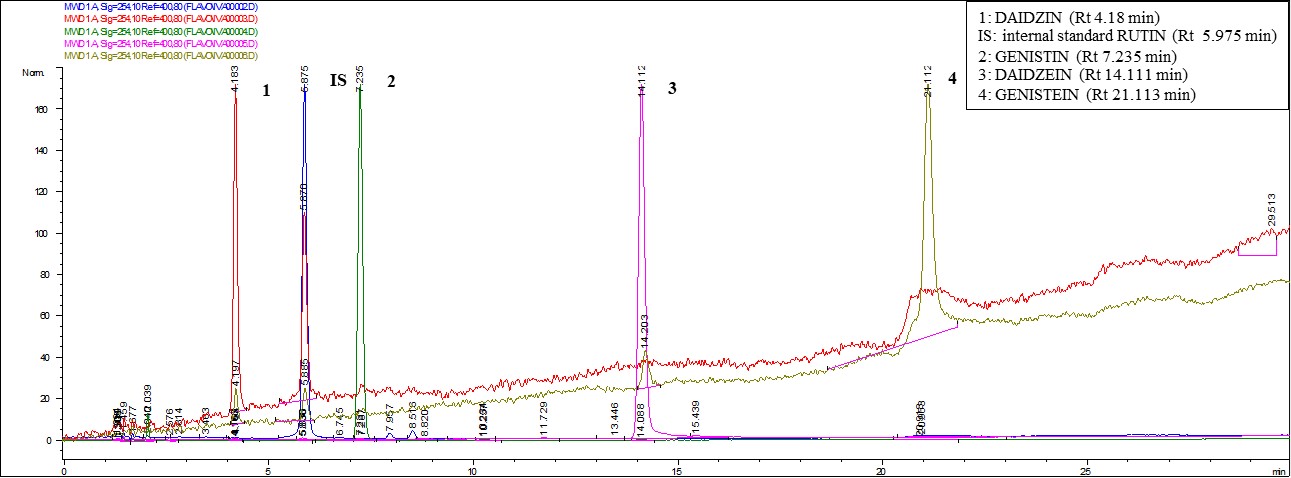


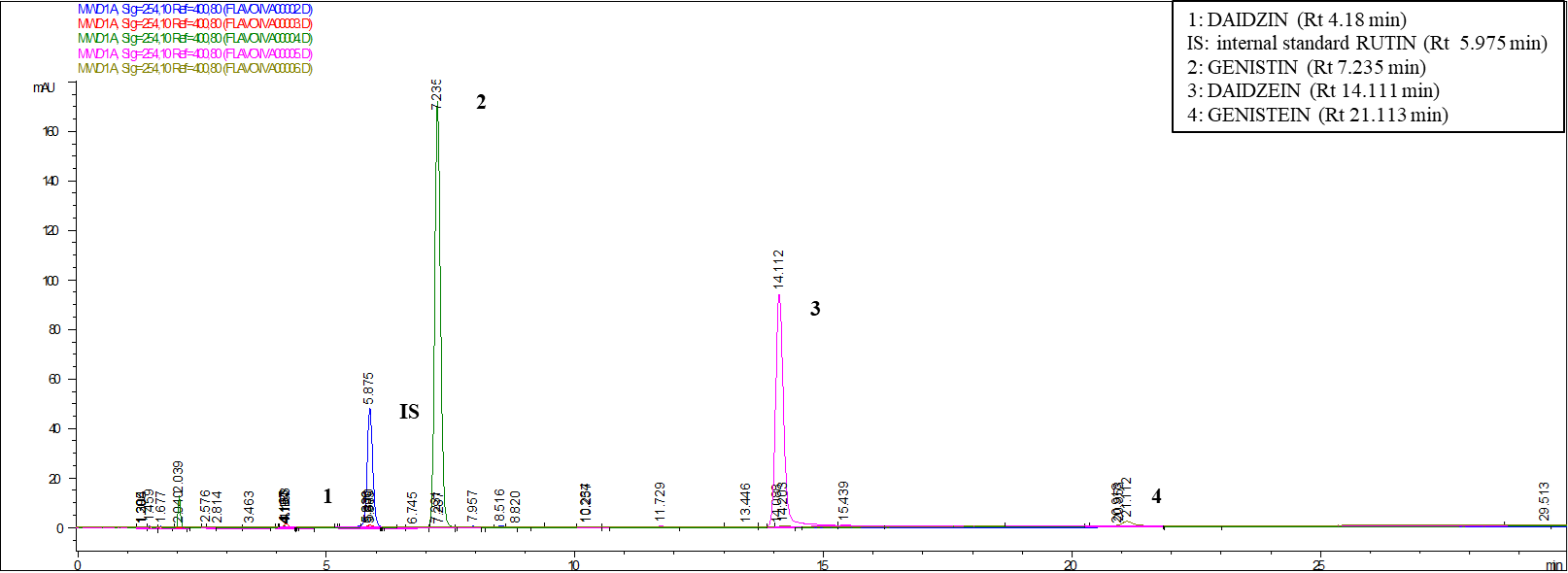


**DAVIS CHROMATOGRAM EXAMPLE: SOYBEAN SEEDS EXTRACTS: CONTROL (BLUE) AND HERBIVORY (RED)**

**
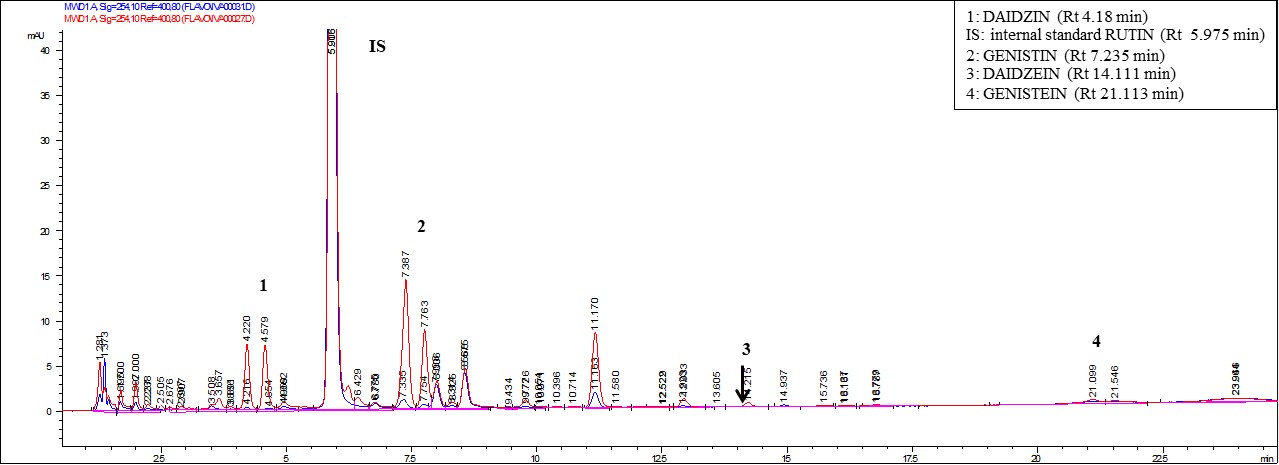
**

**IAC-100 CHROMATOGRAM EXAMPLE: SOYBEAN SEEDS EXTRACTS: CONTROL (BLUE) AND HERBIVORY (RED)**

**
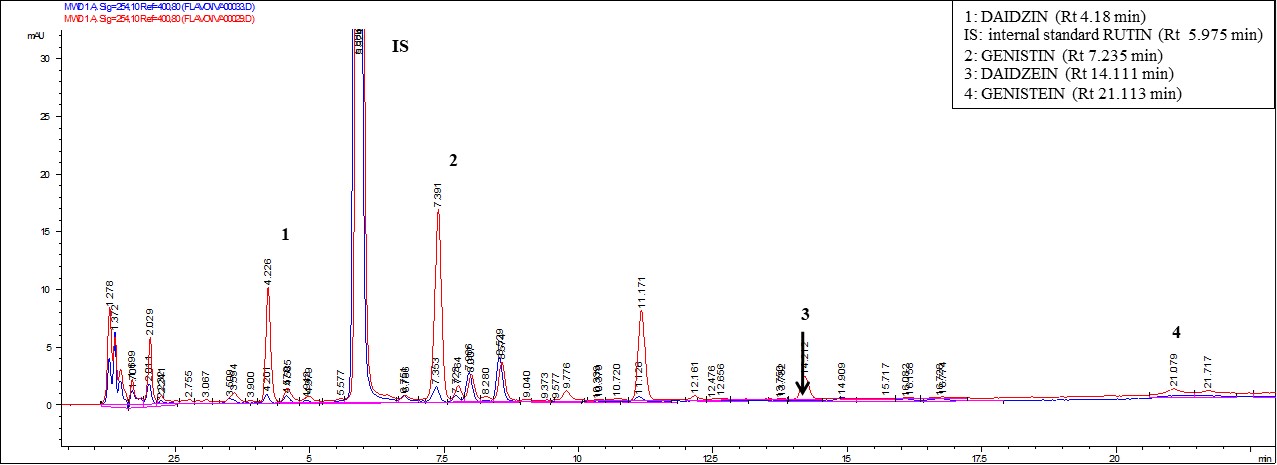
**
